# Assessment of Salivary ABO Blood Group Antigens and Secretor Status in Sriganganagar, Rajasthan: A Correlational Analysis of 300 Samples

**DOI:** 10.7759/cureus.37415

**Published:** 2023-04-11

**Authors:** Gaurav Rajawat, Karthikeyan Ramalingam, Rajat Pareek, Gagandeep Singh, Harleen Narula, Atul Aggarwal

**Affiliations:** 1 Oral Pathology and Microbiology, Surendera Dental College and Research Institute, Sriganganagar, IND; 2 Oral pathology, Saveetha Dental College and Hospitals, Saveetha Institute of Medical and Technical Sciences, Chennai, IND; 3 Oral and Maxillofacial Surgery, Surendera Dental College and Research Institute, Sriganganagar, IND; 4 Oral and Maxillofacial Surgery, Nayar Heart and Multispeciality Hospitals, Amritsar, IND; 5 Pediatric Dentistry, Pacific Dental College and Hospitals, Udaipur, IND

**Keywords:** non-secretors, secretors, saliva, rhesus group, abo blood groups

## Abstract

Aim

To estimate the ABO blood groups from saliva samples and to correlate with the secretor status.

Materials and methods

A sample size of 300 individuals was selected from the outpatient department of Surendera Dental College & Research Institute, Sriganganagar, India, and from dental camps organized by the college in the near vicinity. Informed consent was obtained from selected individuals to collect their blood and saliva samples. Salivary samples were evaluated for ABO blood groups by the absorption-inhibition method. The indicator erythrocytes were prepared after blood group confirmation from serum. It was used to identify the blood group antigens in saliva to confirm the secretor status. The results were tabulated and the Pearson's chi-squared test was performed for statistical analysis using SPSS 15.0 (SPSS Inc., Chicago, IL).

Results

The present study showed that 282 subjects (94%) were Rhesus positive and 18 subjects (6%) were Rhesus negative. Two-hundred-and-fifty subjects (83.3%) were secretors of antigens in saliva. Non-secretors were 50 subjects (16.7%). We identified that 250/300 were secretors and the majority were in AB & A group.

Conclusion

Blood groups could not be detected from the saliva of subjects who were non-secretors. In contrast, blood types could be accurately identified from the saliva of those subjects who were secretors of antigen.

## Introduction

Blood is one of the most crucial pieces of biological evidence in medico-legal situations. Once the blood group is determined, it does not alter over time [1]. The term blood group is applied to inherited antigens detected on the red cell surfaces by specific antibodies [2]. International Society of Blood Transfusion has listed over 300 antigens constituting 33 blood group systems. Yet, ABO and Rh groups are widely used in clinical situations like cross-matching for transfusion and transplantation. ABO is also very important in forensic investigations [1].

Due to the weak antigens and lack of corresponding antibodies in their natural state, other blood groups are less significant. Blood group substances could be identified in diverse body fluids, which are further determined by whether the individual is a secretor or not. A secretor is an individual who releases blood group antigens into bodily fluids and secretions like saliva, mucus, and sweat. The non-secretors do not express blood group antigens in the body secretions. It is also important to note that blood type does not influence the secretor status [1].

Absorption-inhibition approach is utilized in scenarios of blood stain identification. The absorption-elution method can be used to identify ABO blood group components when the sample is of lesser quantity [3,4]. Our study was planned to identify the ABO groups from saliva samples and to correlate with the secretor status of individuals by using the absorption-inhibition method.

## Materials and methods

A sample size of 300 individuals was selected from the outpatient department of Surendera Dental College & Research Institute, Sriganganagar, as well as from dental camps organized by the college in the near vicinity. Patients reporting to the college outpatient department reporting for routine dental procedures were included, whereas uncooperative patients and patients with known systemic diseases were excluded from the study. Informed consent was obtained from selected individuals to collect their blood and saliva samples. This procedure was also cleared by the Institutional Ethical Committee vide letter - SDCRI/IEC/2014/006.

**Figure 1:**
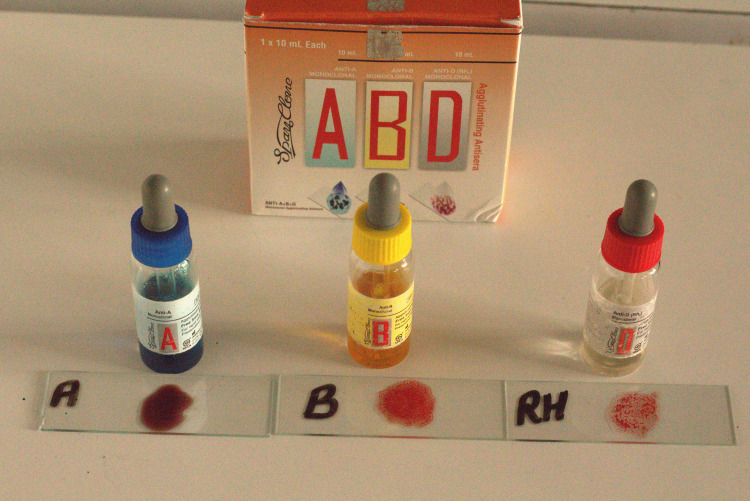
Agglutination reaction of blood samples Picture shows the agglutination reaction of the blood sample on the glass slides after adding the SPAN Monoclonal Agglutinating Antisera (Arkray Healthcare Pvt Ltd, Surat, Gujarat, India)

Peripheral blood samples were collected using the finger prick method and the samples were evaluated using SPAN Monoclonal Agglutinating Antisera (Arkray Healthcare Pvt Ltd, Surat, Gujarat, India) to determine the ABO blood group. Indicator erythrocytes were required for testing salivary samples. Hence, two milliliters of blood were obtained from the cephalic vein of the right arm. Collected blood was centrifuged for 5 minutes at 3000 rpm to separate the red blood cells. The separated erythrocytes were added to 1 ml of saline to prepare the RBC suspension. Simultaneously, the blood group was also determined before salivary analysis (Figure 1).

**Figure 2:**
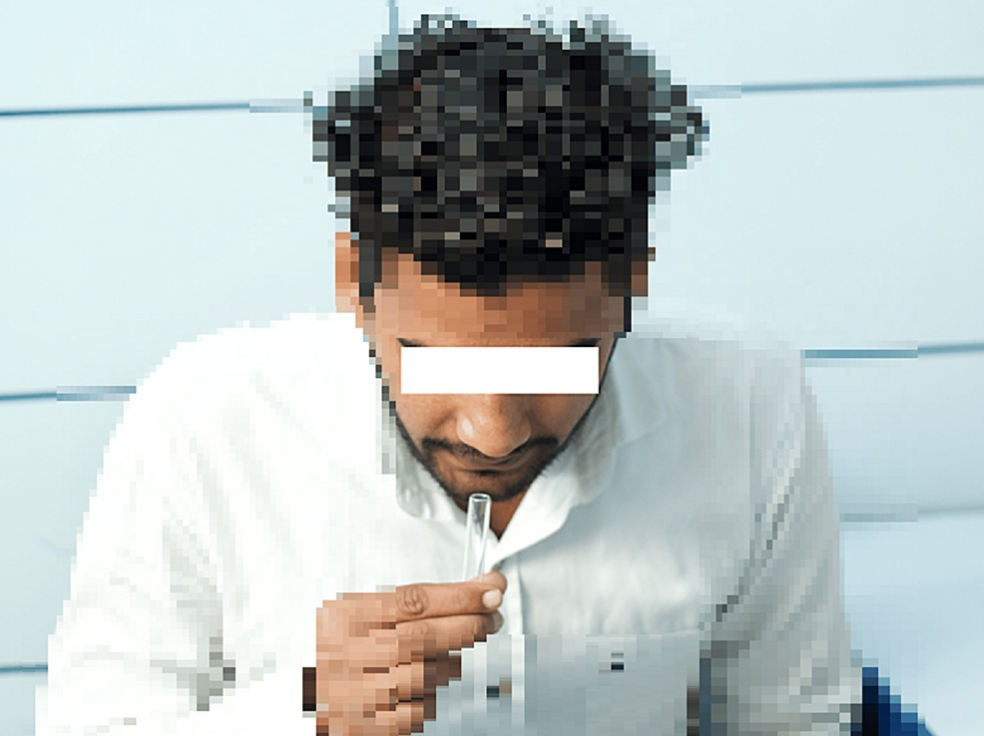
Saliva collection Picture showing the saliva collection from the participant

Oral debris were removed by complete rinsing with water. Approximately 0.5 ml of unstimulated whole saliva samples were collected in clean and dry test tubes (Figure 2).

**Figure 3:**
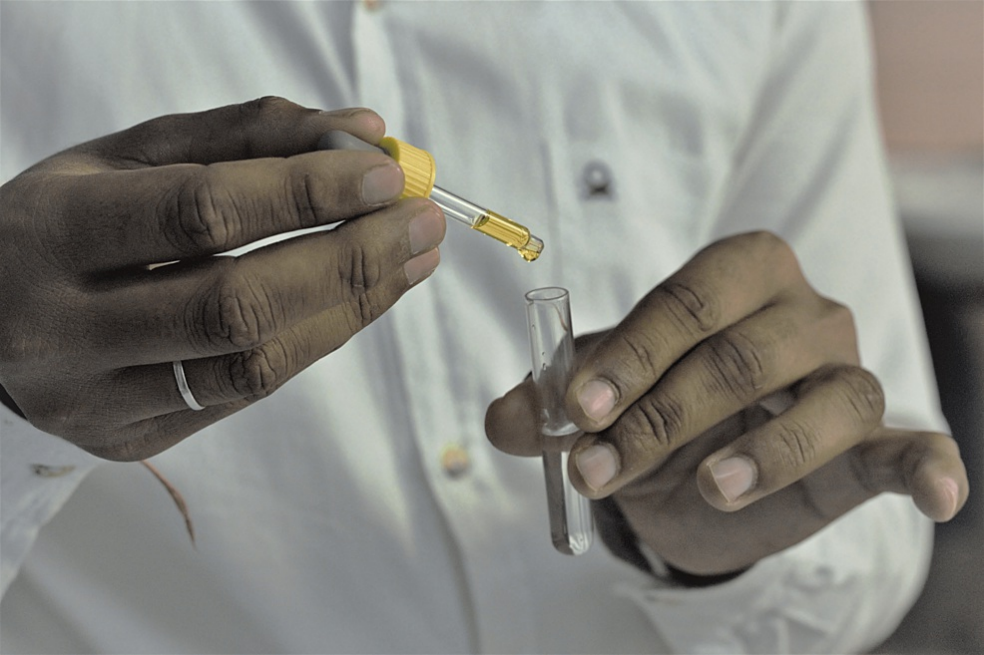
Addition of Anti-sera to the salivary sample Picture showing the addition of SPAN Monoclonal Agglutinating Antisera to the centrifuged salivary sample

The ABO group was determined by the absorption-inhibition method using SPAN Monoclonal Agglutinating Antisera. A Saliva stock solution of 2 ml was prepared by adding distilled water to the collected salivary samples. The samples were placed for 10 minutes in a boiling water bath and then cooled before centrifugation at 3000 rpm for 10 more minutes. The supernatant was discarded and clear saliva was pipetted out from the test tubes. The remaining sample was transferred to another test tube and one drop of diluted antisera (1:4) was added to the test sample based on the previously determined blood group of the subject (Figure 3).

**Figure 4:**
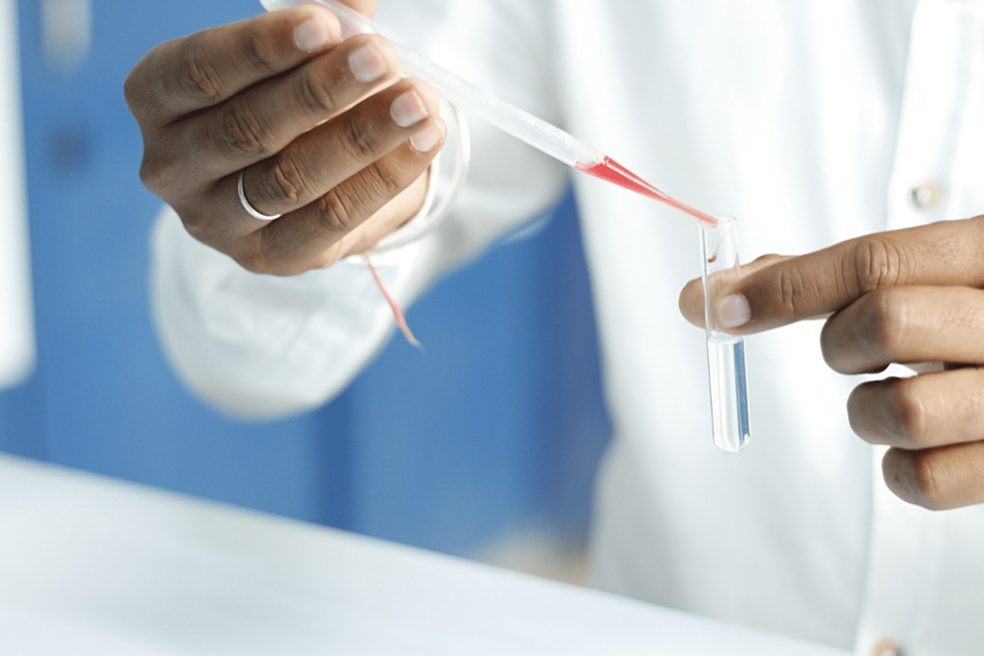
Addition of indicator erythrocytes Picture showing the addition of indicator erythrocytes to the centrifuged salivary sample

One drop of the suitable indicator red cells was introduced into the saliva-antisera mixture. It was mixed properly and left undisturbed for half an hour (Figure 4). Relevant control test tubes were used to monitor the over-dilution of antisera and control test tubes were analyzed with saline instead of salivary sample.

**Figure 5:**
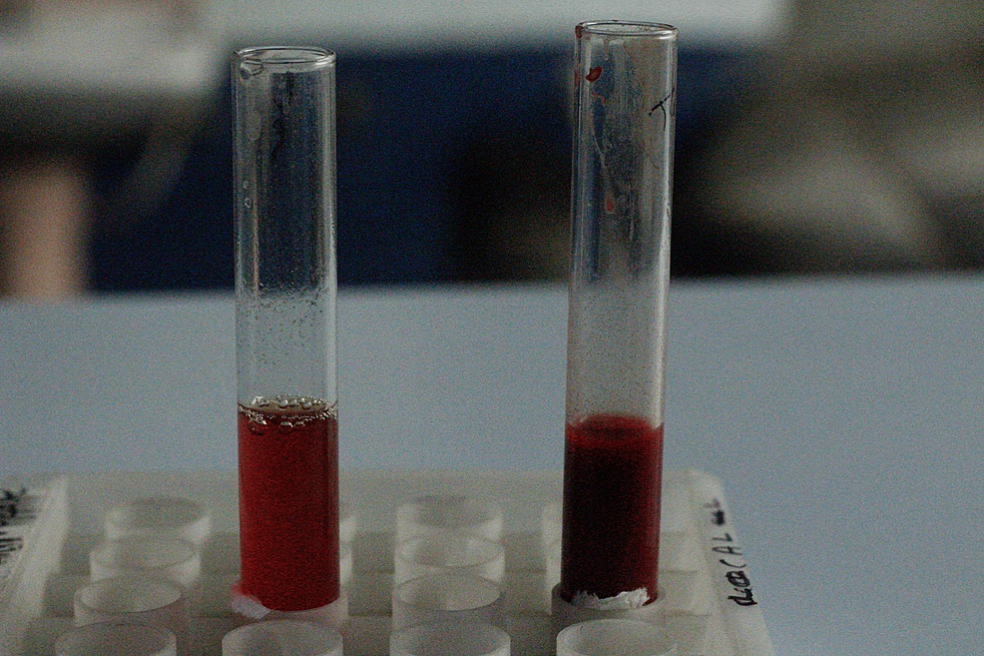
Agglutination reaction Picture showing negative agglutination in salivary sample of secretors (left) and positive agglutination of salivary sample of non-secretors (right)

If there was no agglutination, the entire process was repeated to confirm that it was negative agglutination. Positive agglutination was noted as clumping of erythrocytes in all control test tubes (Figure 5).

**Figure 6:**
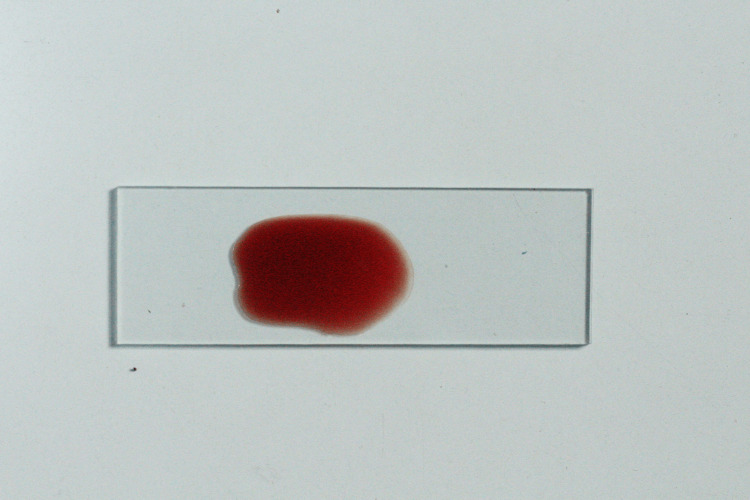
Negative agglutination Picture of the glass slide showing negative agglutination of the salivary sample indicating the secretor status

In the absence of clumping, it was considered a positive result as the salivary blood group antigens had reacted with anti-sera and no more antibody was available to react with red cells and reveal the blood group (Figure 6).

**Figure 7:**
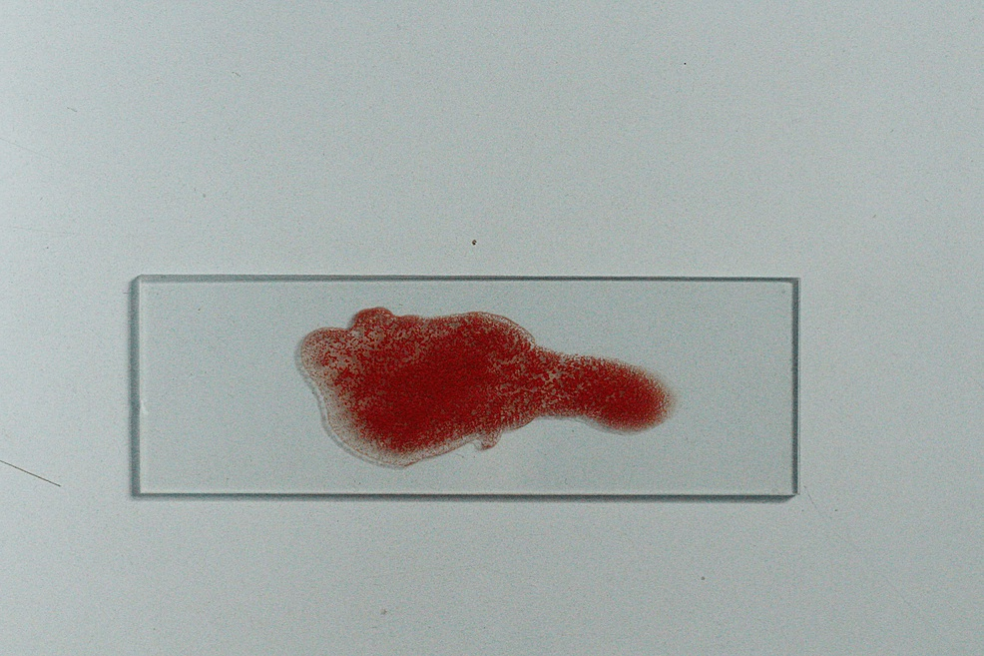
Positive agglutination Picture of the glass slide showing positive agglutination of salivary sample of non-secretors and control samples

The salivary samples that showed clumping of erythrocytes indicated a negative result i.e., the non-secretor status of the tested antigen and it was also noted in our control samples (Figure 7).

The results were tabulated and analyzed with Pearson chi-squared tests using SPSS 15.0 (SPSS Inc., Chicago, IL) software.

## Results

This study was performed among the patients reporting to Surendera Dental College & Research Institute and dental camps conducted by the college in the vicinity of Sriganganagar, Rajasthan, India. Their salivary samples were collected and analysed for ABO blood group antigens and Rhesus group. Correlation between secretor and non-secretor status was determined. Three-hundred participants were included in the study. The age group of participants were between 18-74 years. Their mean age was 35.02 years, with a standard deviation of 11.25 years. Out of 300 subjects, 174 were males, and 126 were females. Males were 58% of the study sample, and females were 42% of the study group.

Serum samples of the study patients revealed A+, A-, AB+. AB-, B+. B-, O+ and O- groups. Thirty-three percent of our samples were O positive, 28% were B positive, 21.7% were A positive, and 11.3% were AB positive. Eight cases were O negative, five cases were A negative, four cases were B negative, and one case was AB negative. The various blood groups of our study are depicted in Table 1.

**Table 1:** ABO and Rhesus blood group of our study Table depicting the distribution of ABO and Rhesus blood group among our study samples

Blood group	No. of cases	Percentage
A-	5	1.7
A+	65	21.7
AB-	1	0.3
AB+	34	11.3
B-	4	1.3
B+	84	28.0
O-	8	2.7
O+	99	33.0
Total	300	100.0

Among the study samples, the majority were Rhesus-positive (282 subjects, 94%). 18 subjects were Rhesus negative (6%) (Table 2).

**Table 2:** Rhesus status This table depicts the distribution of Rhesus positive and Rhesus negative groups among the study samples

Blood group	No. of cases	%age
Rhesus Positive	282	94.00
Rhesus Negative	18	6.00
Total	300	100.00

The present study showed that 250 subjects (83.3%) were secretors of antigen in saliva. Non-secretors were 50 subjects (16.7%) (Table 3).

**Table 3:** Secretor and Non-secretors in Saliva Table showing the distribution of secretors and non-secretors in saliva among the study participants

Saliva sample	No. of cases	%age
Secretors	250	83.30
Non-secretors	50	16.70
Total	300	100.00

Out of 107 subjects of the O group, 91 were secretors and 16 were non-secretors. Out of 88 subjects in the B group, 65 were secretors, and 23 were non-secretors. Out of 70 subjects of the A group, 61 were secretors, and nine were non-secretors. Out of 35 subjects of the AB group, 33 were secretors and two were non-secretors (Table 4).

**Table 4:** Secretor status and blood group comparison Table depicting the comparison between various blood groups and secretor/non-secretor status of our study participants

Blood group	Secretor status		Total	Percentage
	No	Yes		
A-	-	5	5	87.14%
A+	9	56	65	
AB-	-	1	1	94.28%
AB+	2	32	34	
B-	1	3	4	73.3%
B+	22	62	84	
O-	1	7	8	85.04%
O+	15	84	99	
Total	50	250	300	

It was further observed that people with the AB blood group had the maximum secretor percentage of 94.28% (Table 4). Out of 35 subjects, 33 were secretors and two were non-secretors. Out of the total subjects having blood group A, 87.14% were secretors. Out of the total subjects having blood group B, 73.3% were secretors, and 85.04% of subjects having blood group O were secretors. Out of 250 secretors, 234 were Rhesus positive and 16 were Rhesus negative. Among 50 non-secretors, 48 subjects were Rhesus positive and two were Rhesus negative. Similarly, 12.9% of the A group, 26.1% of the B group, 5.7% of the AB group, and 15% of the O group were non-secretors. The Pearson chi-squared test among the ABO blood groups, Rhesus groups, and secretor status showed significant results with a p-value of 0.022 (x2 value = 9.662; df = 3; likelihood ratio = 0.019).

## Discussion

Apart from blood, these antigens could be secreted into numerous body fluids including saliva. So the presence or absence of antigens in saliva or other body fluids, such as semen, urine, sweat etc., determines that a person is a secretor or non-secretors [4]. Blood groups are utilized in medico-legal investigations as it remains unaltered in every individual, and if a secretor, the relevant blood group could be identified accurately from saliva [5].

Motghare et al. analyzed the population from Maharashtra [1], while Metgud et al. evaluated the population from Southern Rajasthan [4] for salivary blood group estimation. Our study was performed on a population of Sriganganagar which is in North-Western Rajasthan.

In our study of 300 subjects, 83.3% were secretors, out of which 174 were males and 126 were females. Motghare et al. conducted a study with 200 patients, out of which 101 were males and 99 were females [1]. Metgud et al. have reported 99% accuracy in their study [4]. Sharma et al. conducted a large study to assess the secretor status [6]. Tabasum et al. conducted a study in which 90 samples were collected from patients [7].

Gilmiyarova et al. found that out of 89 healthy subjects, 42.5% had blood group 0(I), 31.5% had blood group A(II), 16.5% had blood group B(III), and 9.5% had blood group AB(IV), with Rh-positive 82.7% and Rh-negative 17.3% [8]. Tejasvi et al. conducted a similar study on 60 healthy individuals which showed 86.6 % of the subjects were secretors while the remaining 13.33 were non-secretors [9]. Saliva of non-secretors will always yield negative results and require further testing to identify the appropriate blood groups.

The secretor status could be identified by two methods - the absorption-inhibition method (ab-in) and the absorption-elution method. Ruth et al. conducted a study to determine blood groups from cigarette butts [10]. Velani et al. conducted research on 47 dry salivary samples and showed that 100% of the subjects were secretors [11]. Both studies utilized the absorption-inhibition method.

Yu-Hua et al. conducted a study to determine the distribution of h type 1 and h type 2 antigens of the ABO blood group in different cells of the human submandibular gland in which they used conventional hemagglutination techniques using mouse MAbs against histo-blood group antigens [12].

Situations are noted when blood is not found at the site of investigation. As the majority of the population are secretors of blood group antigens in saliva, proper handling of salivary samples from multiple items at the crime scene is critical to identify the suspects [13].

Salivary blood group antigens could be used for the preliminary elimination of suspects during forensic investigations [14]. Future applications of newer approaches utilizing natural active compounds and nanoparticles could be possible [15-18].

Further studies are needed to ascertain the reliability of ABO blood group antigens from other bodily secretions like sweat. The mucosal concentration of relevant substances could give a clue about the systemic diseases of the individual [19].

Limitations

The ABO blood grouping using saliva samples depends upon the secretor status of a person. As discussed in our study, around 83.3 % of subjects were secretors and this also implies that we can not determine the blood groups of the remaining 16.7 % of subjects using the absorption-inhibition method. If a person is not a secretor, then this method of determining blood groups has limited use.

## Conclusions

Salivary sample collection could be used in special situations where blood sampling is not possible due to co-morbidities or at times of emergency or disasters. We observed that a majority of our study samples were secretors of blood group antigens in their saliva and could be used as a reliable marker for identification. Multiple studies have revealed that the majority of people are secretors and can be used in crime scene investigations, where more advanced and sensitive techniques are not available. Very few studies are reported on the Indian population about the ABO blood group systems and further studies are required to confirm the same in diverse conditions.
